# Dioxygenase JID1 mediates the modification of OPDA to regulate jasmonate homeostasis

**DOI:** 10.1038/s41421-023-00530-6

**Published:** 2023-04-11

**Authors:** Rong Yi, Ran Du, Jiaojiao Wang, Jijun Yan, Jinfang Chu, Jianbin Yan, Xiaoyi Shan, Daoxin Xie

**Affiliations:** 1 Tsinghua-Peking Center for Life Science, and MOE Key Laboratory of Bioinformatics, School of Life Sciences, Tsinghua University, Beijing, China; 2 College of Agronomy, Inner Mongolia Agricultural University, Hohhot, China; 3 Shenzhen Branch, Guangdong Laboratory for Lingnan Modern Agriculture, Key Laboratory of Synthetic Biology, Ministry of Agriculture and Rural Affairs, Chinese Academy of Agricultural Sciences, Shenzhen, Guangdong China; 4 National Centre for Plant Gene Research, Institute of Genetics and Developmental Biology, Chinese Academy of Sciences, Beijing, China; 5 College of Advanced Agricultural Sciences, University of Chinese Academy of Sciences, Beijing, China

**Keywords:** Jasmonic acid, Plant molecular biology

Dear Editor,

The phytohormone jasmonate (JA), including jasmonic acid and its oxylipin derivatives, is essential for plant resistance against various stresses^1,2^. The biosynthetic pathway of JA is initiated from α-linolenic acid (18:3) that is converted to 12-oxo-phytodienoic acid (OPDA) by 13-Lipoxygenase (LOX), Allene Oxide Synthase (AOS), and Allene Oxide Cyclase (AOC), which is then reduced to OPC-8:0 by OPDA Reductase3 (OPR3) and activated by OPC-8:0 CoA Ligase1 (OPCL1) followed by three cycles of β-oxidation catalyzed by acyl-CoA Oxidase (ACX), Multifunctional Protein (MFP), and 3-ketoacyl-CoA Thiolase (KAT) to yield jasmonic acid. Jasmonic acid is further catalyzed by Jasmonate Resistant1 (JAR1) to generate jasmonoyl-l-isoleucine (JA-Ile)^1^. Excess jasmonic acid and JA-Ile are hydroxylated by Jasmonate-Induced Oxygenases (JOX1, JOX2, JOX3, and JOX4) that belong to 2-oxoglutarate/Fe(II)-dependent dioxygenase (2OGD) superfamily, and cytochrome P450 enzymes (CYP94B3, CYP94B1, and CYP94C1) for the regulation of JA homeostasis, respectively^3,4^, thereby driving the growth-defense tradeoff in plants.

OPDA is not only the first cyclic precursor of JA biosynthesis but also an independent signaling molecule^5,6^. OPDA coordinates with or without the canonical JA pathway to regulate a unique subset of genes and thereby orchestrates plant defense against pathogen infection and insect attack^5,7^. In response to stress, accumulated OPDA binds cyclophilin 20-3 to form a complex with Serine Acetyltransferase 1, which facilitates the formation of a hetero-oligomeric cysteine synthase complex with O-acetylserine (thiol) lyase B to stimulate cysteine production, thereby shifting cellular redox potentials. The cellular redox capacity is then enhanced to activate a subset of OPDA-responsive genes responsible for pathogen defense and stress adaptation^8^.

Despite extensive research on the biological properties and biosynthesis of OPDA, none of the plant enzymes involved in OPDA catabolism has been reported so far. Here, we identify *Jasmonate-Induced Dioxygenase1* (*JID1*) that encodes a 2OGD modifying OPDA and reducing the conversion of OPDA into jasmonic acid and JA-Ile. We further demonstrate that the catabolism of OPDA by JID1 serves as an important mechanism to fine-tune the JA homeostasis essential for plant defense responses.

To search enzymes responsible for OPDA catabolism in the JA pathway (Fig. 1a), we analyzed the available JA-regulated transcriptome data (TAIR Accession: 1007965964)^9^ using a series of screening parameters (Supplementary Fig. S1a). Eighteen oxygenase genes yielded from such a search approach, caught our eyes straight away as 14 of those genes have been reported to encode enzymes for hormonal biosynthesis or catabolism^3,10,11^ (Supplementary Fig. S1b). The remaining four genes (*AT1G06620*, *AT3G61400*, *CYP705A12*, and *CYP81D1*) with unknown biochemical functions were selected for further analysis by ATTED-II CoExSearch database. Only *AT1G06620* was shown to tightly co-express with several JA biosynthetic or catabolic genes, as well as genes encoding the key components in JA signaling (Supplementary Fig. S1c), implying a possible role for *AT1G06620* in JA catabolism.

**Fig. 1:**
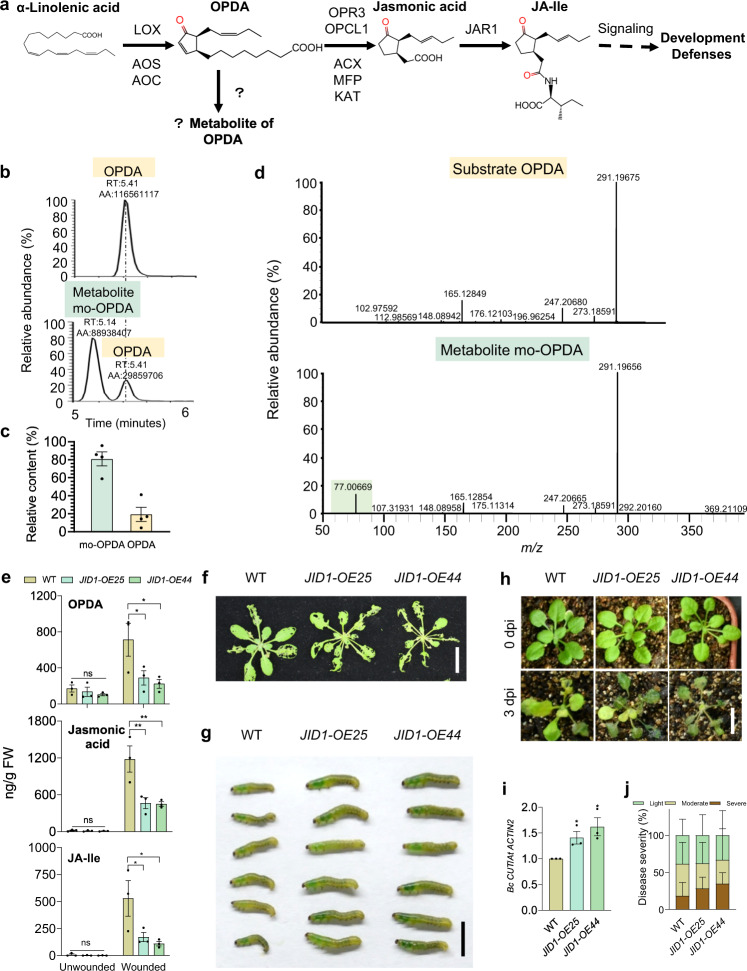
JID1 modifies OPDA to regulate plant defense responses. **a** Schematic of JA biosynthesis. Enzymes involved in JA biosynthesis are indicated. The enzyme(s) involved in the catalysis of OPDA is unclear. **b** LC-MS/MS analysis of residual OPDA and produced mo-OPDA after incubation with empty vector (top panel) or affinity-purified JID1 protein (bottom panel) respectively, monitored by UHPLC-QE/MS. RT, retention time; AA, peak area. **c** Relative contents of substrate OPDA and product mo-OPDA after incubation with JID1 protein. Relative content was calculated by the ratio of residual OPDA or produced mo-OPDA peak area relative to that of initial OPDA. The initial content of OPDA was set as 100%. Data are means ± SEM (*n* = 4). **d** MS/MS spectra of substrate OPDA (top panel) and product mo-OPDA (bottom panel) monitored by UHPLC-QE/MS. The *m/z* 77.00669 fragment of mo-OPDA is indicated in green. **e** JA profiles of plants without (Unwounded) or with mechanical wounding (Wounded) for 1 h. Data are means ± SEM (*n* = 3). Two-way ANOVA, Tukey’s post-hoc test, ns, *P* > 0.05, *P ≤ 0.05, **P ≤ 0.01. **f**, **g** Representative phenotypes of attacked plants (**f**) and *S. exigua* larvae (**g**) are shown. Scale bars, 2 cm (**f**), 5 mm (**g**). **h**–**j** Disease symptoms of plants (**h**), quantification of *B. cinerea* biomass (**i**), and disease severity of plants (**j**) are shown. Scale bar, 1 cm (**h**). Data are means ± SEM (*n* = 3). Student’s *t*-test, *P ≤ 0.05. dpi, days post-inoculation.

Phylogenetic analysis showed that *AT1G06620* belongs to the DOXC clade of 2OGD superfamily (Supplementary Fig. S2a). DOXC emerges as an essential regulator in phytohormone homeostasis through oxidation or hydroxylation of active hormones, such as JA, gibberellic acid (GA), salicylic acid (SA), and indole-3-acetic acid (IAA)^10^. *AT1G06620* gene, which encodes a 2OGD with an N-terminal non-heme dioxygenase domain (PF14226) and a C-terminal 2OGD domain (PF03171) (Supplementary Fig. S2b, c), was significantly induced upon the treatment with OPDA (Supplementary Fig. S2d) or methyl jasmonate (MeJA) (Supplementary Fig. S2e). In contrast, neither OPDA nor MeJA could obviously induce the expression of three homologous genes (*AT3G61400*, *AT1G03400*, and *AT1G03410*) grouping into the same subclade DOXC31 with *AT1G06620* (Supplementary Fig. S2d, e). *AT1G06620* was therefore termed as *Jasmonate-Induced Dioxygenase1* (*JID1*), which is the prime candidate with a possible role in the JA catabolic pathway.

To observe the tissue-specific expression of *JID1*, we generated the transgenic plants harboring *pJID1::GUS*. In the 8-day-old seedlings, strong expression of GUS was detected in the cotyledon, leaf, hypocotyl, as well as root tip, but relatively weak expression was observed in the primary root (Supplementary Fig. S3a). In adult plants, GUS expression occurred predominately in the vascular tissue of the leaf (Supplementary Fig. S3b), which is similar to the expression patterns of JA biosynthetic genes, such as *AOS*, *AOC*, and *LOX*. In addition, GUS activity was mainly found in the sepal, filament, and base of mature silique (Supplementary Fig. S3c). Further real-time PCR analysis displayed that the *JID1* transcript abundance was highest in the root, followed by the flower, stem, and leaf, whereas the lowest expression was detected in the mature silique (Supplementary Fig. S3d).

To investigate the subcellular localization of JID1, we first transiently expressed the JID1-GFP fusion construct in *Nicotiana benthamiana* (*N. benthamiana*) leaf epidermal cells. Compared with the uniform distribution of GFP-empty vector throughout the cell, JID1-GFP was localized in the nucleus stained by DAPI and cytoplasm, whereas it did not colocalize with the chloroplast autofluorescence or peroxisome marker mCherry-Peroxisome Targeting Signal Type1 (PTS1) (Supplementary Fig. S4a, b). The dual localization was further confirmed by immunoblot analysis of JID1-GFP in cytosolic and nuclear fractions (Supplementary Fig. S4c) and fluorescence results in leaves of *Arabidopsis* transgenic plants stably expressing p35S::JID1-GFP (Supplementary Fig. S4d). Considering that several members of the 2OGD involved in phytohormone biosynthesis or catabolism have dual cytoplasmic/nuclear localization^12^, we hypothesized that cytoplasm and nucleus are potential sites for JID1-mediated JA catabolism.

To gain insights into the induced dynamic expression pattern of *JID1*, we subjected *Arabidopsis* plants to the treatment with MeJA, wounding, or pathogen, and analyzed the *JID1* transcription levels at different time points. After MeJA treatment, the expression of *JID1* was significantly increased with the maximum at the late stage (9.48 folds at 6 h) (Supplementary Fig. S5a), which is similar to the expression pattern of jasmonic acid oxygenase *JOX2*^4^. As a control, the expression of *OPR3* was quickly induced at an early stage (21.14 folds at 1 h) but decreased afterward (Supplementary Fig. S5a). Furthermore, *JID1* exhibited a peak expression at 3 h after wounding treatment in a JA receptor Coronatine Insensitive1 (COI1)-dependent manner (approximately ninefold enhancement relative to the untreated control) (Supplementary Fig. S5b). Similarly, *Botrytis cinerea* (*B. cinerea*) infection caused a sustained increase of *JID1* expression in WT plants during the 24-h treatment (Supplementary Fig. S5c). The induced expression with a peak at the late stage upon the treatment with MeJA, wounding, or pathogen is compatible with a possible function of JID1 in JA catabolism.

To determine the enzymatic activity of JID1, recombinant JID1 was first purified from *Escherichia coli* (*E. coli*) expression system. However, no catalytic activity was detected for such JID1 purified from the prokaryotic expression system (Supplementary Fig. S6). As the prokaryotic expression system may attenuate the activity of DOXC31 subclade enzymes^13^, we next transiently expressed *JID1* in *N. benthamiana* leaves and affinity-purified JID1 protein for enzymatic analysis (Supplementary Fig. S7). The JID1 with high purity from the eukaryotic expression system was incubated with OPDA, jasmonic acid, JA-Ile, or MeJA, then monitored by UHPLC-TSQ/MS. Notably, incubation with JID1 significantly reduced the OPDA level, while the level of jasmonic acid, JA-Ile, or MeJA was unchanged (Supplementary Fig. S8).

The mass spectra of the JID1 enzymatic product were further characterized by UHPLC-QE/MS. After incubation with JID1, the OPDA (retention time 5.41 min) level decreased by about 80%, and a new metabolite (*m/z* 369.21109, retention time 5.14 min), termed as modified-OPDA (mo-OPDA), was obviously accumulated (Fig. 1b, c). Further MS/MS results showed that such metabolite mo-OPDA had the main characteristic OPDA fragments and a new fragment of *m/z* 77.00669 (Fig. 1d), which suggests that mo-OPDA is a product derived from OPDA. These results collectively suggest that the JID1 enzyme specifically catalyzes OPDA into mo-OPDA. Due to the unavailability of mo-OPDA, it is technically difficult to determine its structural formula. Further characterization of the exact catalytic product will improve our understanding of the JID1-mediated OPDA catabolic pathway.

To define the enzymatic activity of JID1 in vivo, we generated transgenic plants overexpressing Flag*-*tagged JID1 (Supplementary Fig. S9) and measured the levels of OPDA, jasmonic acid, and JA-Ile in these plants. After mechanical wounding, the OPDA level in WT was triggered to 712.37 ng/g fresh weight (FW) (Fig. 1e). In *JID1-OE25* and *JID1-OE44* plants, OPDA levels were detected at 289.24 and 223.04 ng/g FW respectively, much less than that in WT (Fig. 1e). Similarly, the wound-induced jasmonic acid levels in *JID1-OE25* and *JID1-OE44* plants were severely attenuated to only 39.33% (464.55 ng/g FW) and 38.12% (450.27 ng/g FW) of that in WT plants (1181.24 ng/g FW), respectively (Fig. 1e). Wound-induced JA-Ile contents in *JID1-OE25* (171.73 ng/g FW) and *JID1-OE44* plants (110.85 ng/g FW) were also significantly reduced compared with WT control (529.08 ng/g FW) (Fig. 1e). These data demonstrate that overexpression of *JID1* decreases OPDA level and thereby reducing the contents of jasmonic acid and JA-Ile.

In light of the catabolic activity of JID1 on OPDA, we next asked whether JID1 could attenuate plant defense responses. We first examined the effect of *JID1* overexpression on wound-induced gene expression. In WT plants, as expected, expression of two OPDA-responsive genes, *CYP81D11* and *GRX480*^7,8^, as well as two JA-responsive genes, *VSP1* and *JAZ10*, was significantly upregulated after wounding treatment (Supplementary Fig. S10a, b). In accordance with the low levels of OPDA and JA-Ile (Fig. 1e), wound-induced expression of these four genes was obviously repressed in *JID1-OE25* and *JID1-OE44* plants (Supplementary Fig. S10a, b). These results suggest that overexpression of *JID1* downregulates wounding responses by reduction of OPDA level and the conversion of OPDA into JA-Ile.

Consistent with the impaired induction on the wound-induced expression of defense-related genes (Supplementary Fig. S10a, b), *JID1-OE25* and *JID1-OE44* plants were more susceptible to *Spodoptera exigua* (*S. exigua*) feeding compared with WT control (Fig. 1f). The larvae of *S. exigua* fed with *JID1-OE25* and *JID1-OE44* plants were larger and weighed significantly more (~1.83- and 1.57-fold, respectively) compared with those fed on WT plants (Fig. 1g; Supplementary Fig. S10c). When challenged with fungal pathogen *B. cinerea*, *JID1-OE25* and *JID1-OE44* plants displayed severe susceptibility, as revealed by the more serious disease symptoms, higher *B. cinerea* levels (~1.41- and 1.62-fold *B. cinerea* genomic *CUTINASE* DNA, respectively), and increased plant disease severity in comparison with WT plants (Fig. 1h–j). These data collectively suggest that overexpression of *JID1* negatively regulates JA-mediated plant defense responses.

Interestingly, the amounts of OPDA, jasmonic acid, and JA-Ile in loss-of-function mutants *jid1-1* and *jid1-5* were similar to those of WT (Supplementary Figs. S9a, b, S11a). Moreover, the wound-induced expression of *GRX480*, *CYP81D11*, *VSP1*, and *JAZ10* was hardly affected in *jid1-1* and *jid1-5* mutants (Supplementary Fig. S11b, c). Consistently, no detectable changes in defense responses against herbivory were observed between WT control and *jid1* mutants (Supplementary Fig. S11d–f). Similar to the previous observation that several enzymes act redundantly in the biosynthesis or catabolism of phytohormones including IAA^14^ and JA^4^, our data suggest that some unidentified genes might function redundantly with *JID1* to mediate the catabolism of OPDA (Supplementary Fig. S11). Further study on these functionally redundant genes would shed insight into OPDA catabolic pathway.

In conclusion, we identify a 2OGD that specifically modifies OPDA and reveals a missing OPDA catabolic mechanism to regulate JA homeostasis. Moreover, we provide novel insights into the function of OPDA catabolism in the regulation of plant defense responses. The homeostasis between biosynthesis and catabolism of defense-related phytohormone JA plays a pivotal role in fine-tuning the growth-defense tradeoff. As the upstream player in the JA catabolic pathway, the expression of *JID1* is rapidly induced in response to mechanical wounding and pathogen infection (Supplementary Fig. S5b, c). JID1-mediated modification of OPDA could prevent overaccumulation of JA and subsequently attenuate plant defense responses (Fig. 1; Supplementary Fig. S10), defining an important role of JID1 in regulating JA homeostasis essential for the growth-defense tradeoff.

## Supplementary information

Supplementary information
