# Supplementary material for: Dioxygenase JID1 mediates the modification of OPDA to regulate jasmonate homeostasis
Source: Cell Discov. 2023 Apr 11;9:39. doi: 10.1038/s41421-023-00530-6 (PMC10090039; doi:10.1038/s41421-023-00530-6)
Supplement: Supplementary file 1 — Supplementary information [file 41421_2023_530_MOESM1_ESM.pdf]

a

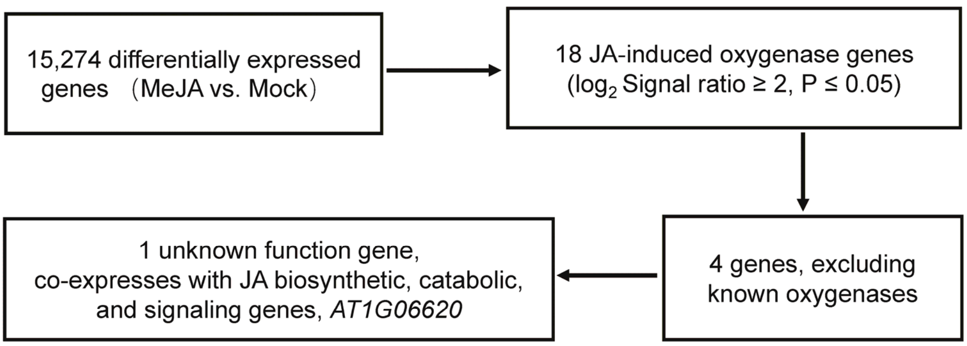

b

	No.	AGI No.	Gene annotation	Function
	1	AT1G72520	LOX4, Lipoxygenase 4	JA biosynthesis
	2	AT2G27690	CYP94C1, cytochrome P450, family 94, subfamily C, polypeptide 1	JA hydroxylation
	3	AT1G17420	LOX3, Lipoxygenase 3	JA biosynthesis
	4	AT5G05600	JOX2, 2-oxoglutarate and Fe(II)-dependent oxygenase superfamily protein	JA hydroxylation
	5	AT5G63450	CYP94B1, cytochrome P450, family 94, subfamily B, polypeptide 1	JA hydroxylation
	6	AT5G52320	CYP96A4, cytochrome P450, family 96, subfamily A, polypeptide 4	Fatty acid hydroxylatior
	7	AT2G38240	JOX4, 2-oxoglutarate and Fe(II)-dependent oxygenase superfamily protein	JA hydroxylation
	8	AT4G10500	S3H, 2-oxoglutarate and Fe(II)-dependent oxygenase superfamily protein	SA hydroxylation
	9	AT5G42650	AOS, cytochrome P450, family 74, subfamily A	JA biosynthesis
	10	AT1G14130	DAO1, 2-oxoglutarate and Fe(II)-dependent oxygenase superfamily protein	IAA oxidation
*	11	AT1G06620	similar to a 2-oxoglutarate-dependent dioxygenase	unknown
	12	AT1G14120	DAO2, 2-oxoglutarate and Fe(II)-dependent oxygenase superfamily protein	IAA oxidation
	13	AT3G22400	LOX5, Lipoxygenase 5	JA biosynthesis
*	14	AT3G61400	1-aminocyclopropane-1-carboxylate oxidase-like protein	unknown
*	15	AT5G42580	CYP705A12, cytochrome P450, family 705, subfamily A, polypeptide 12	unknown
*	16	AT5G36220	CYP81D1, cytochrome P450, family 81, subfamily D, polypeptide 1	unknown
	17	AT3G01900	CYP94B2, cytochrome P450, family 94, subfamily B, polypeptide 2	JA hydroxylation
	18	AT2G22330	CYP79B3, cytochrome P450, family 79, subfamily B, polypeptide 3	IAA biosynthesis

c

No.	AGI No.	Gene annotation	Pathway
1	AT5G05600	JOX2, 2-oxoglutarate and Fe(II)-dependent oxygenase superfamily protein	JA catabolism
2	AT3G25780	AOC3, Allene Oxide Cyclase 3	JA biosynthesis
3	AT2G06050	OPR3, 12-oxo-phytodienoate Reductase 3	JA biosynthesis
4	AT5G13220	JAZ10, Jasmonate-Zim-Domain protein 10	JA signaling
5	AT1G67560	LOX6, Lipoxygenase 6	JA biosynthesis
6	AT1G74950	JAZ2, Jasmonate-Zim-Domain protein 2	JA signaling
7	AT1G44350	ILL6, IAA-Leucine Resistant (ILR)-Like Gene 6	JA catabolism
8	AT1G30135	JAZ8, Jasmonate-Zim-Domain protein 8	JA signaling
9	AT1G32640	MYC2, Jasmonate Insensitive 1	JA signaling
10	AT2G27690	CYP94C1, cytochrome P450, family 94, subfamily C, polypeptide 1	JA catabolism
11	AT1G19180	JAZ1, Jasmonate-Zim-Domain protein 1	JA signaling
12	AT1G20510	OPCL1, OPC-8:0 CoA Ligase 1	JA biosynthesis

Fig. S1: Identification of *AT1G06620.*

a Screening for oxygenase genes involved in JA catabolism.

b List of 18 highly expressed oxygenase candidates induced by JA. Asterisks denote 4 genes with unknown biochemical functions.

c List of *AT1G06620* co-expressed genes involved in JA biosynthetic, catabolic, and signaling pathways using ATTED-II CoExSearch (http://atted.jp).

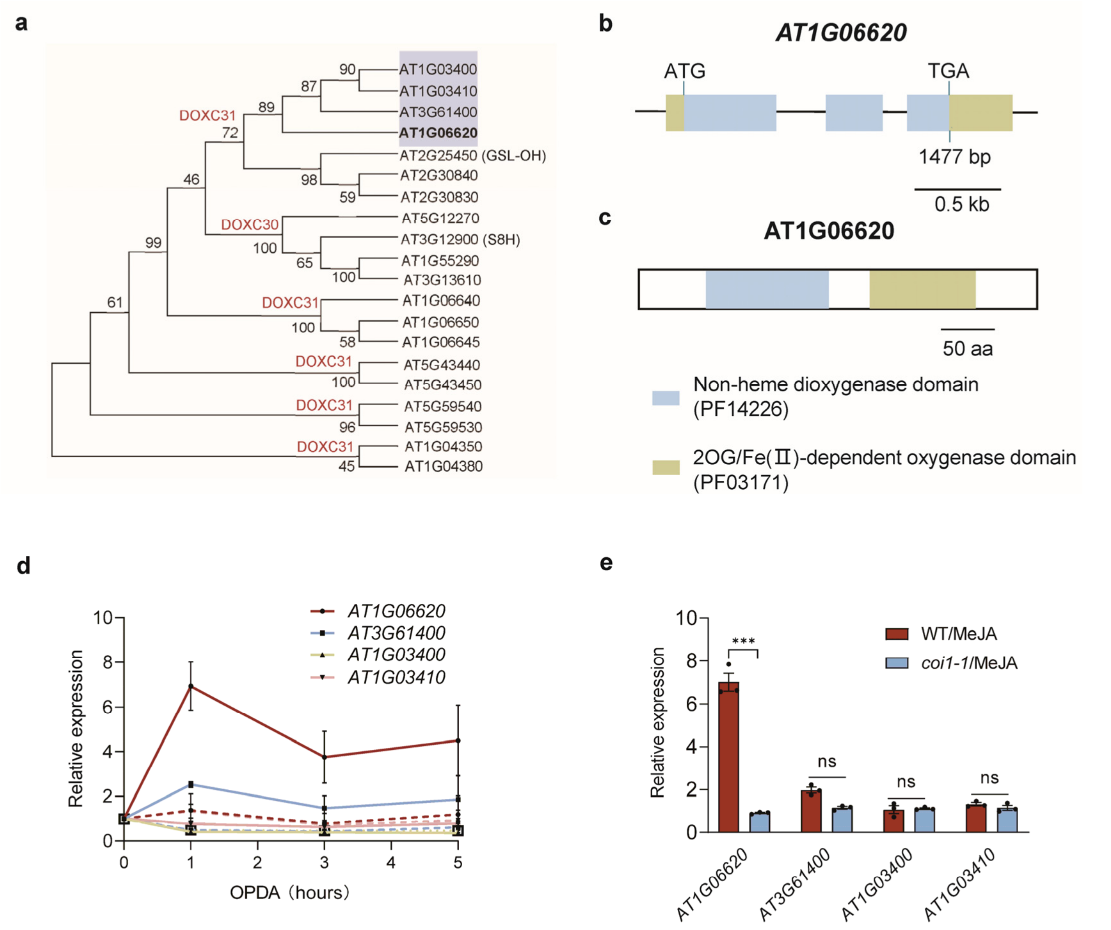

Fig. S2: Identification of AT1G06620 as a potential oxygenase in JA catabolic pathway.

a Phylogenetic tree of AT1G06620 and related 2OGDs in *Arabidopsis*. Enzymes with known biochemical functions are labeled with names. AT1G06620 and its homologs are indicated in blue.

b Diagram of *AT1G06620* gene. The blue frame, yellow frame, and dark line denote the CDS, 5'- or 3'-UTR, and intron of *AT1G06620*, respectively. Scale bar, 0.5 kb.

c Diagram of AT1G06620 protein. The blue frame and yellow frame denote the N-terminal non-heme dioxygenase domain (PF14226) and the C-terminal 2-oxoglutarate and Fe(II)-dependent oxygenase domain (PF03171), respectively. Scale bar, 50 amino acids (aa).

d Relative expression levels of *AT1G06620*, *AT3G61400*, *AT1G03400*, and *AT1G03410* in 7-day-old WT seedlings treated without (dash lines) or with 15

μM OPDA (solid lines) at indicated time points.

e Relative expression levels of *AT1G06620*, *AT3G61400*, *AT1G03400*, and *AT1G03410* in 7-day-old WT or *coi1-1* seedlings treated with 50 μM MeJA for 6 h. For (**d, e**), *ACTIN2* was used as the internal control. Data are means ± SEM (*n* = 3). For (**e**), data are analyzed by two-way ANOVA, Tukey's post-hoc test, ns, $P > 0.05$, ***$P \leq 0.001$.

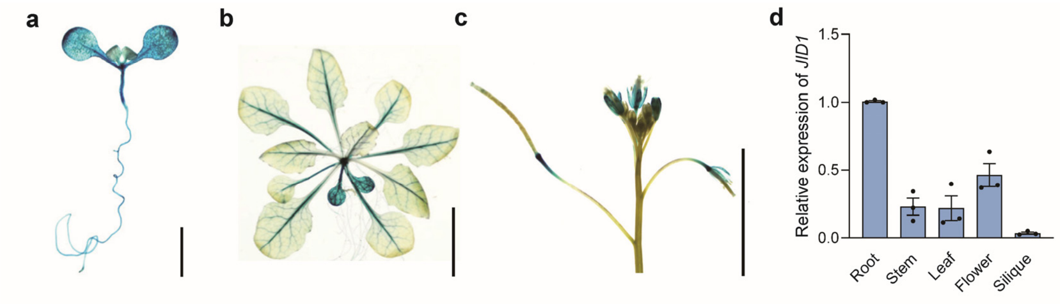

Fig. S3: Expression patterns of *JID1*.

a–**c** Histochemical GUS staining of 8-day-old seedling (**a**), 4-week-old plant grown under SD conditions (**b**), and inflorescence of plant (**c**) expressing *pJID1::GUS*. Scale bars, 5 mm (**a**); 1 cm (**b, c**).

d Relative expression levels of *JID1* in different tissues (root, stem, leaf, flower, and silique) of 5-week-old WT plants. *ACTIN2* was used as the internal control. Data are means ± SEM (n = 3).

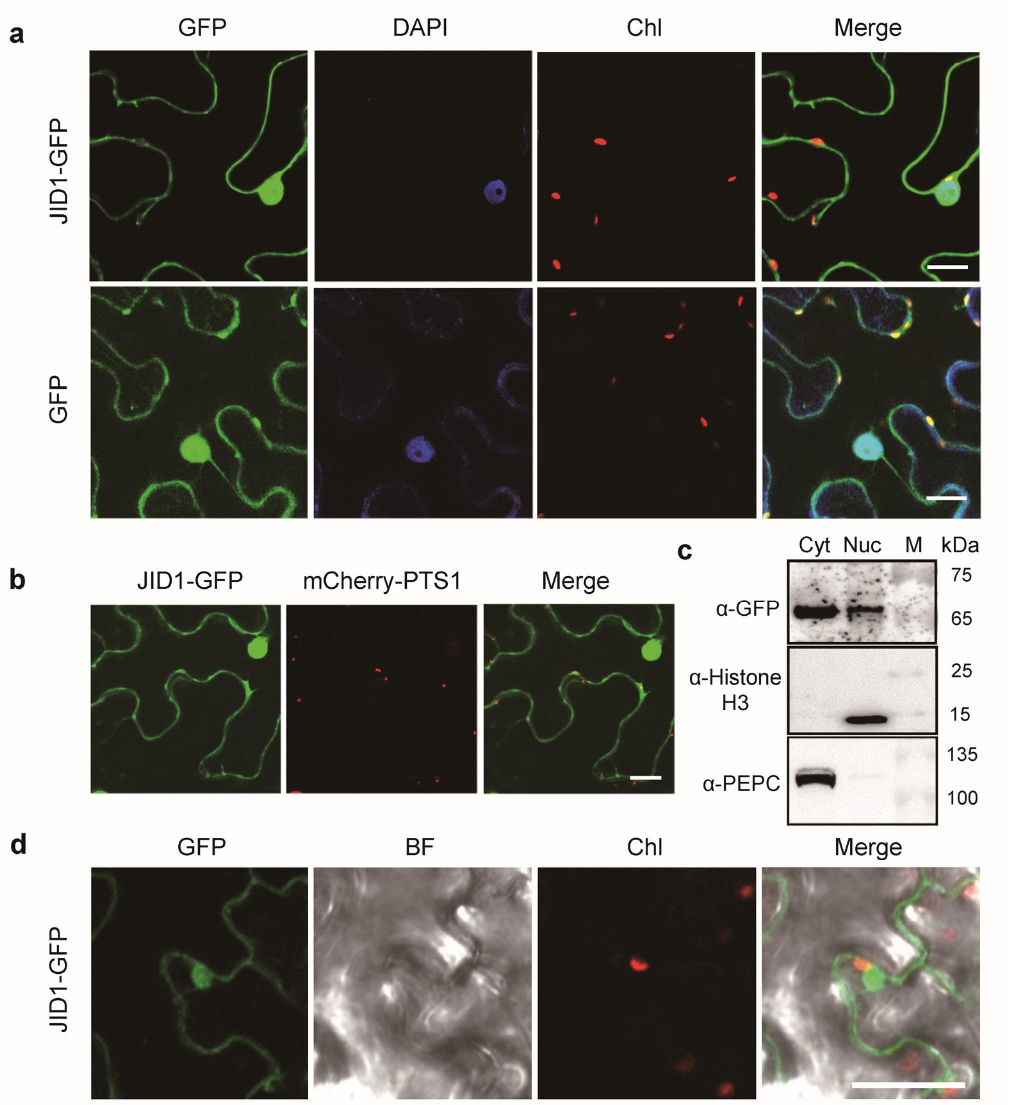

Fig. S4: Expression analysis of JID1.

a, b Confocal images of JID1-GFP (or GFP) with DAPI (**a**), chloroplast autofluorescence (Chl) (**a**), and mCherry-PTS1 (**b**) in epidermal cells of *N. benthamiana* leaves. Scale bars, 20 μm.

c Immunoblot analysis of JID1 subcellular localization. Total proteins extracted from *N. benthamiana* leaves transiently expressing *JID1-GFP* were fractioned. JID1-GFP proteins in the cytoplasm (Cyt) and nucleoplasm (Nuc) fractions were analyzed by immunoblot using GFP antibody. Cytoplasmic proteins were ten-fold diluted. Histone H3 and PEPC were used as nuclear and cytosolic markers, respectively. M, molecular weight ruler (kDa).

d Confocal images of JID1-GFP in leaf epidermal cells of 7-day-old *Arabidopsis* seedlings stably expressing *p35S::JID1-GFP*. BF, bright field. Scale bar, 20 μm.

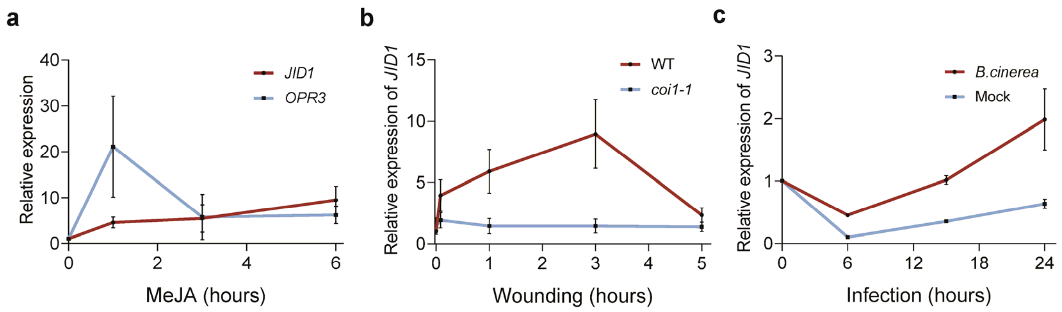

Fig. S5: Expression analysis of *JID1* under MeJA, wounding, or *B. cinerea* infection.

a Relative expression levels of *JID1* and *OPR3* in 7-day-old WT seedlings treated with 25 μM MeJA at indicated time points.

b Relative expression levels of *JID1* in 4-week-old WT and *coi1-1* plants grown under SD conditions upon mechanical wounding at indicated time points.

c Relative expression levels of *JID1* in 3-week-old WT plants grown under SD conditions after spray inoculation with potato dextrose broth (Mock) or *B. cinerea* at indicated time points. For (**a**-**c**), *ACTIN2* was used as the internal control. Data are means ± SEM (*n* = 3).

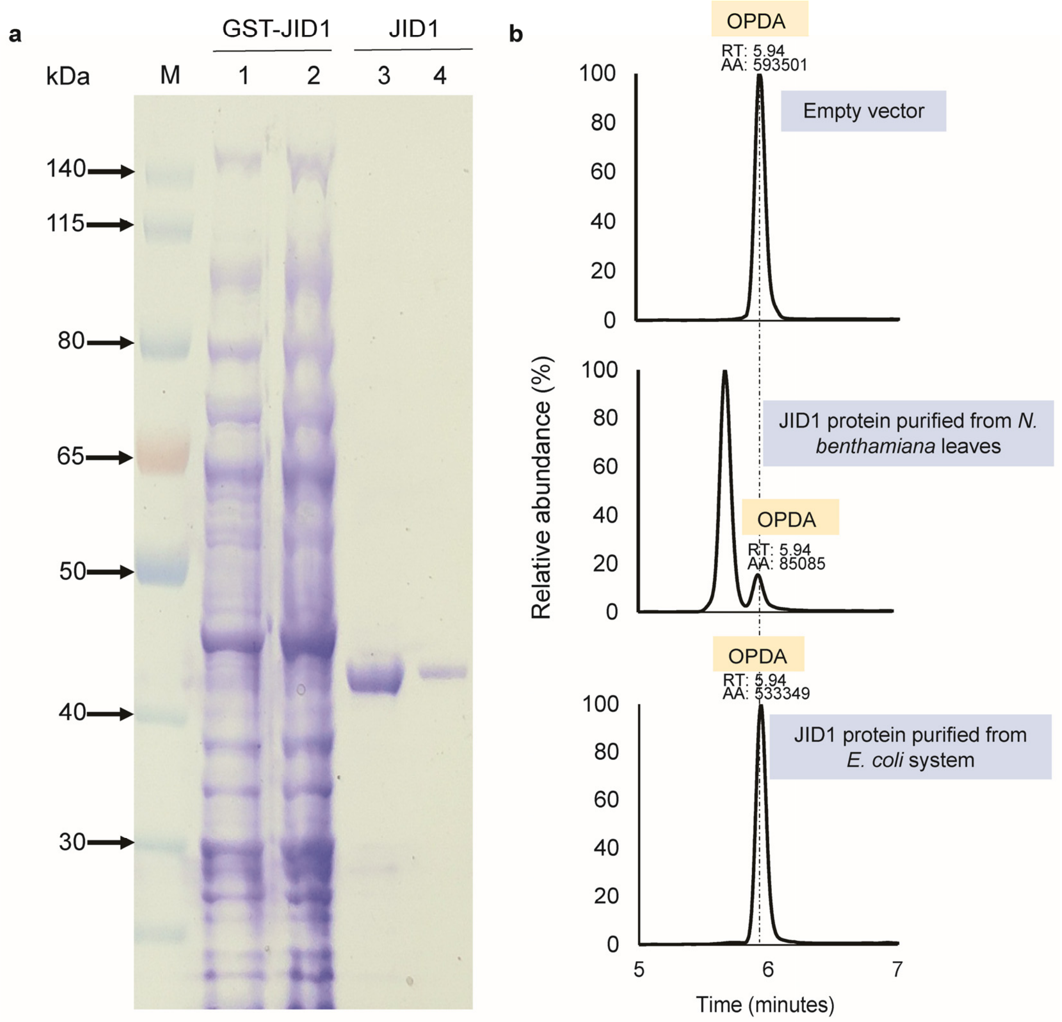

Fig. S6: Purification and catalytic activity analysis of recombinant JID1 protein from *E. coli* expression system.

a Coomassie blue staining of *E. coli*-expressed JID1 protein after IPTG induction. Lane 1,2, cell lysate of IPTG-induced *E. coli* cells harboring *GST-JID1* construct; lane 3,4, purified JID1 protein after on-column cleavage of GST tag by prescission protease from IPTG-induced *E. coli* cells. M, molecular weight ruler (kDa).

b LC-MS analysis of residual OPDA after incubation with empty vector (top panel), affinity-purified JID1 protein from transiently expressed *N. benthamiana* leaves (medium panel, the positive control), or recombinant JID1 protein from *E. coli* expression system (bottom panel) respectively, monitored by UHPLC-TSQ/MS. RT, retention time; AA, peak area.

a

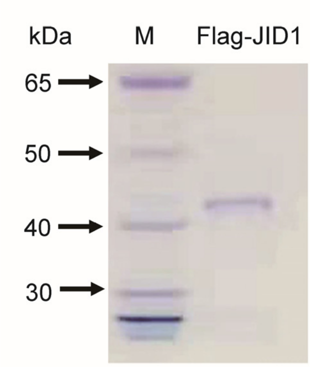

b

No.	Gene name or locus	Gene accession	Score	Coverage
1	AT1G06620.1	similar to a 2-oxoglutarate-dependent dioxygenase	4771.63	85.21
2	Niben101Scf03493g00020.1	ACTIN3	89.02	28.65
3	Niben101Scf01982g01005.1	ACTIN2	88.15	27.85
4	Niben101Scf00173g09004.1	ribulose-bisphosphate carboxylases, large subunit	86.09	14.82
5	Niben101Scf02262g02007.1	basic helix-loop-helix DNA-binding superfamily protein	61.24	6.64
6	Niben101Scf08618g01001.1	elongation factor 1-alpha	14.82	8.60
7	Niben101Scf01478g05007.1	glutamate-1-semialdehyde 2,1-aminomutase 2	12.33	9.75
8	Niben101Scf03243g00006.1	TUBULIN alpha	8.40	13.11
9	Niben101Scf05177g02025.1	glyceraldehyde-3-phosphate dehydrogenase	8.29	8.20
10	Niben101Scf03133g00003.1	glyceraldehyde-3-phosphate dehydrogenase 2	6.44	6.04

Fig. S7: Purification of JID1 from *N. benthamiana* transient expression system.

a, b Coomassie blue staining (**a**) and protein mass spectrometry (**b**) of affinity-purified JID1 protein from transiently expressed *N. benthamiana* leaves. M, molecular weight ruler (kDa).

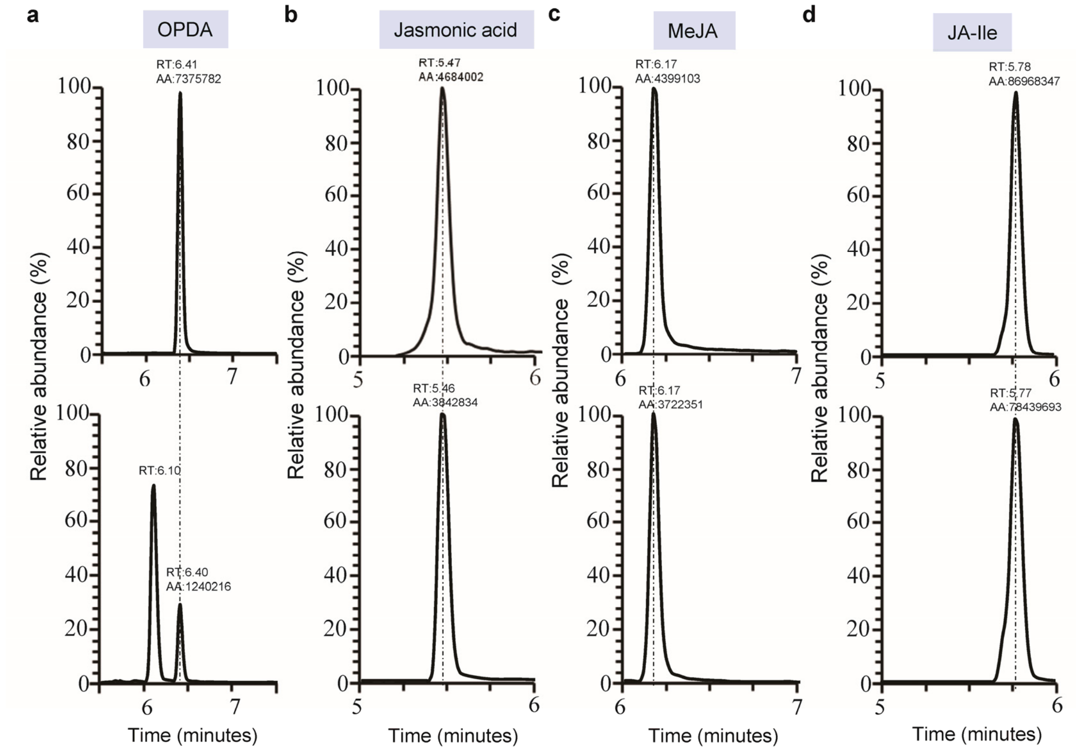

Fig. S8: Consumption of OPDA, jasmonic acid, MeJA, or JA-Ile by affinity-purified JID1 protein *in vitro*.

a–**d** LC-MS analysis of residual OPDA (**a**), jasmonic acid (**b**), MeJA (**c**), or JA-Ile (**d**) after incubation with empty vector (top panel) or affinity-purified JID1 protein (bottom panel) from transiently expressed *N. benthamiana* leaves respectively, monitored by UHPLC-TSQ/MS. RT, retention time; AA, peak area.

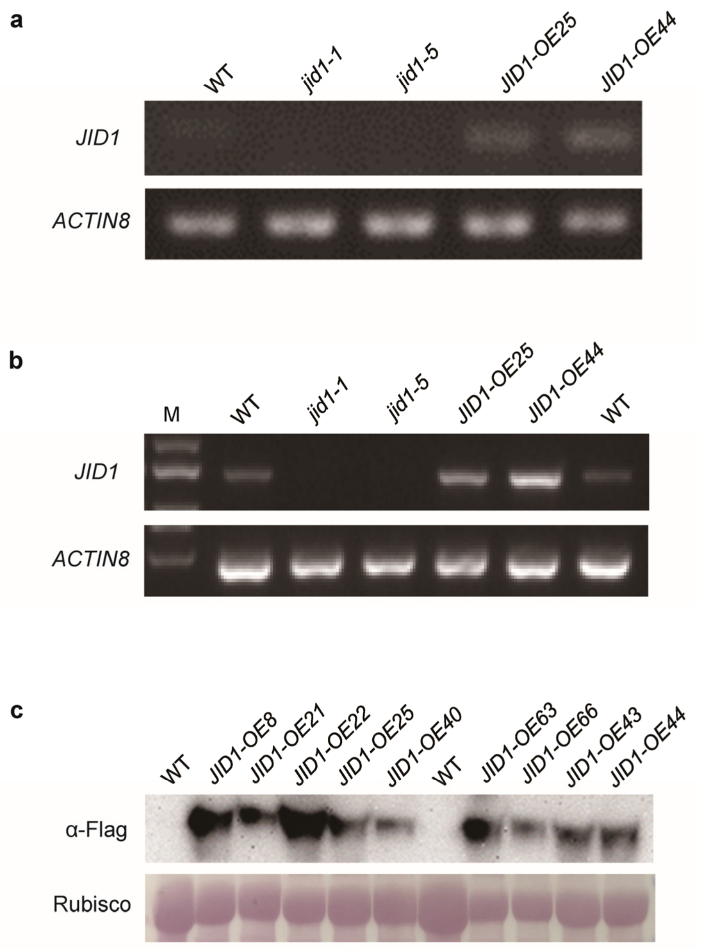

Fig. S9: Identification of *JID1* mutants and overexpression lines.

a,b Semiquantitative RT-PCR analysis of *JID1* in WT, *jid1-1*, *jid1-5*, *JID1-OE25*, and *JID1-OE44* plants at the resting state (**a**) or upon 3 h-mechanical wounding (**b**), respectively. *ACTIN8* was used as the internal control.

c Western-blot analysis of JID1 protein in *JID1* overexpression lines with anti-Flag antibody. The staining of Rubisco was used as a loading control.

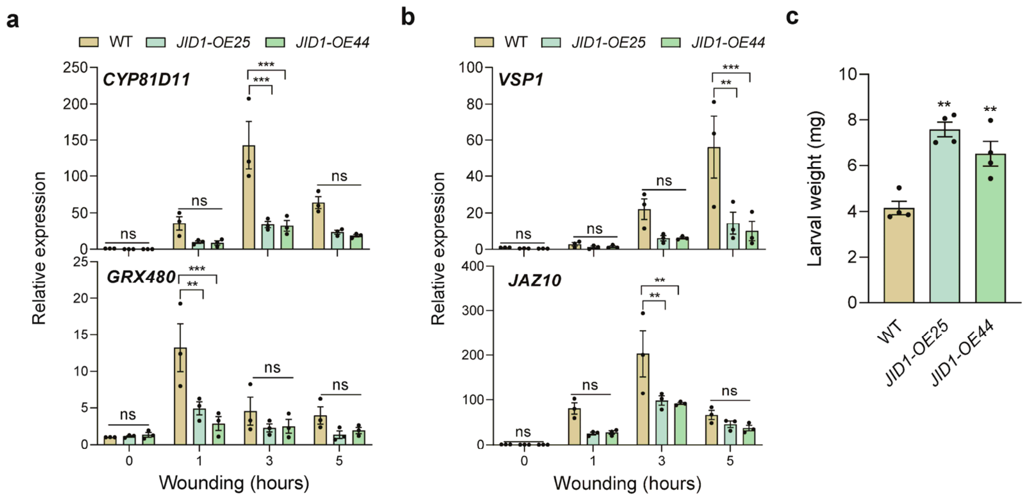

Fig. S10: Attenuated defense responses in *JID1* overexpression lines.

a, b Relative expression levels of OPDA-responsive genes *CYP81D11*, *GRX480* (**a**) and JA-responsive genes *VSP1*, *JAZ10* (**b**) in 4-week-old WT, *JID1-OE25*, and *JID1-OE44* plants grown under SD conditions upon mechanical wounding at indicated time points. *ACTIN2* was used as the internal control. Data are means ± SEM (n = 3). Two-way ANOVA, Tukey's post-hoc test, ns, $P > 0.05$, **$P \leq 0.01$, ***$P \leq 0.001$.

c Average weight of *S. exigua* larvae reared on WT, *JID1-OE25*, and *JID1-OE44* plants. Data are means ± SEM (n = 4). Student's t-test, **$P \leq 0.01$.

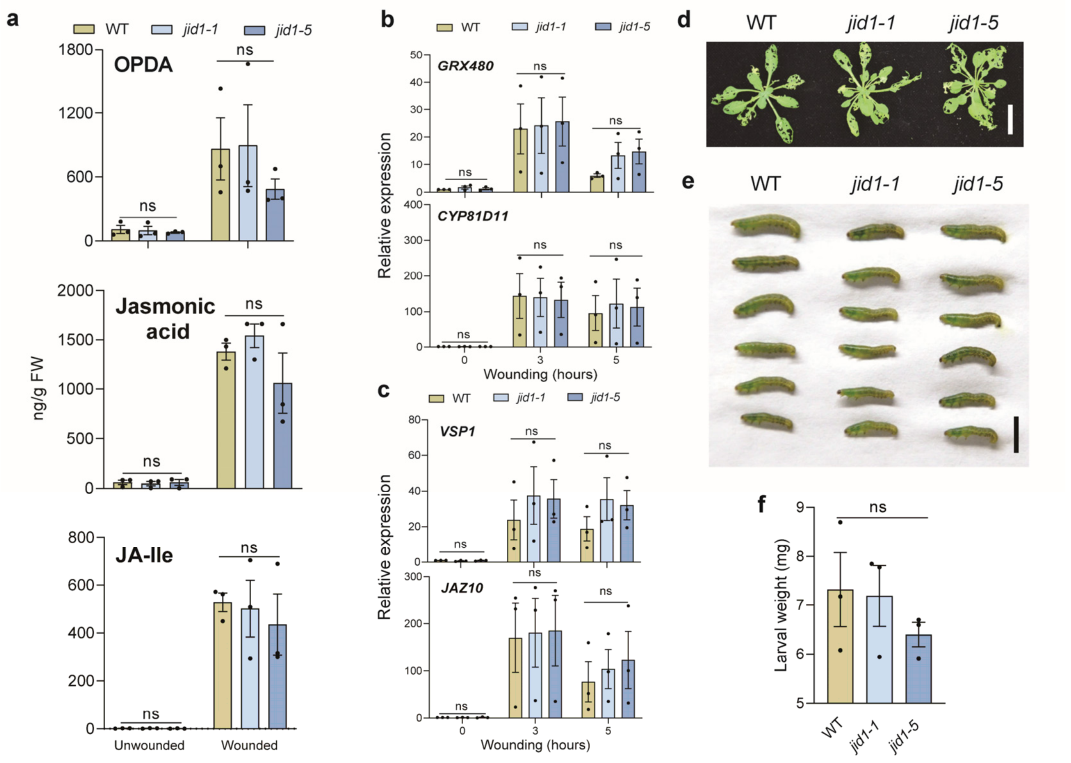

Fig. S11: Defense responses in *JID1* knockout mutants.

a JA profiles of WT, *jid1-1*, and *jid1-5* plants without (Unwounded) or with mechanical wounding (Wounded) for 1 h. OPDA, jasmonic acid, JA-Ile levels were calculated by correcting for the internal standard d_5-JA and plant fresh weight. Data are means ± SEM (*n* = 3). Two-way ANOVA, Tukey's post-hoc test, ns, P > 0.05.

b, **c** Relative expression levels of OPDA-responsive genes *GRX480*, *CYP81D11* (**b**) and JA-responsive genes *VSP1*, *JAZ10* (**c**) in 4-week-old WT, *jid1-1*, and *jid1-5* plants grown under SD conditions upon mechanical wounding at the indicated time points. *ACTIN2* was used as the internal control. Data are means ± SEM (*n* = 3). Two-way ANOVA, Tukey's post-hoc test, ns, *P* > 0.05.

d Representative phenotypes of 4-week-old WT, *jid1-1*, and *jid1-5* plants grown under SD conditions after feeding with *S. exigua* for 3 d. Scale bar, 2 cm.

e Representative phenotypes of *S. exigua* larvae recovered from WT, *jid1-1*, and *jid1-5* plants described in (**d**). Scale bar, 5 mm.

f Average weight of *S. exigua* larvae reared on WT, *jid1-1*, and *jid1-5* plants described in (**d**). Data are means ± SEM (*n* = 3). Student's *t*-test, ns, *P* > 0.05.

Table S1. Primers used in this study.

Name	Orientation	Sequence (5'→3')
ACTIN2-Realtime	Forward	gtggattccagcagcttccat
ACTIN2-Realtime	Reverse	gctgagagattcagatgccca
AT1G06620-Realtime	Forward	tgggttgatgttcctcctg
AT1G06620-Realtime	Reverse	aaccctatgctccacgctta
OPR3-Realtime	Forward	ttggacgcaactgattctgac
OPR3-Realtime	Reverse	gtaggcgtggtagcgaggtt
jid1-1 (SALK_092918)	Forward	tttgatgtgtaaggcccaaag
jid1-1 (SALK_092918)	Reverse	gaaaaatccccatttctctgc
jid1-5 (SALK_095793)	Forward	tgatgggtccataaactcgag
jid1-5 (SALK_095793)	Reverse	ctcctctgatttctccatccc
LBb1.3		attttgccgatttcggaac
AT1G06620pro-GUS	Forward	attacgccaagcttggctgcagaaaataatctgatcatcttgca
AT1G06620pro-GUS	Reverse	ggccagtgaattcccggggatcctgcttttagctgaaaatgcaat
Flag-AT1G06620	Forward	ctgcaggggcccggggtcgacatggagtcatcacttccccaagt
Flag-AT1G06620	Reverse	ctcggtaccggatccactagttcagatcttgagatagagtaatcc
AT1G06620-GFP	Forward	cgggggactctagacgcggatccatggagtcatcacttccc
AT1G06620-GFP	Reverse	agtgaaaagttcttctcctttacccatggtaccgatcttgagatagagtaa
AT1G06620-Semi RT	Forward	tcacagagattccttcgatatttcgtg
AT1G06620-Semi RT	Reverse	tggtcatgaagaacttgaagccc
ACTIN8-Semi RT	Forward	atgaagattaaggtcgtggcac
ACTIN8-Semi RT	Reverse	gtttttatccgagtttgaagaggc
mCherry-PTS1 (Mutagenesis)	Forward	catggacgagctgtacaagtcgaagctgtgatctagactgca
mCherry-PTS1 (Mutagenesis)	Reverse	cagcttcgacttgtacagctcgtccatgccgccggtggagtg
GST-AT1G06620	Forward	ttccaggggcccctgggatccatggagtcatcacttcc
GST-AT1G06620	Reverse	gcggccgctcgagtcgacccgggtcagatcttgagataga
VSP1-Realtime	Forward	acgtccagtcttcggcatcc
VSP1-Realtime	Reverse	tagttgatggacagtccctc
JAZ10-Realtime	Forward	atcccgatttctccggtcca
JAZ10-Realtime	Reverse	actttctccttgcgatgggaaga
GRX480-Realtime	Forward	tgattgtgattggacggaga
GRX480-Realtime	Reverse	taaaccgccggtaacttcac
CYP81D11-Realtime	Forward	cgagaaacgtgtggagaaagt
CYP81D11-Realtime	Reverse	tcccggcaagtatcataacaa
CUTINASE-Realtime	Forward	gatgtgacggtcatctttgccc
CUTINASE-Realtime	Reverse	agatttgagagcggcgagg
AT3G61400-Realtime	Forward	agcaatggcaagttcataagc
AT3G61400-Realtime	Reverse	cggttcagccgctctattt
AT1G03400-Realtime	Forward	tcttgcaagcctgaagtcg
AT1G03400-Realtime	Reverse	gagatcaacggtagggatgg
AT1G03410-Realtime	Forward	atgaccaatgctgggttgat
AT1G03410-Realtime	Reverse	tatgctccgcgctaatgaat

Materials and methods

Plant materials and growth conditions

The *Arabidopsis thaliana* WT, *jid1* mutants, and transgenic plants used in this research were all in Columbia-0 (Col-0) background. Plants were cultured in the growth chamber with 16 h light (21 - 23°C)/8 h dark (16 - 19°C) Long Day (LD) photoperiod. For mechanical wounding, pathogen inoculation, and insect feeding assays, plants were grown in the growth chamber with 8 h light (21 - 23°C)/16 h dark (16 - 19°C) SD photoperiod. The *N. benthamiana* seedlings were cultured in the growth chamber with 16 h light (25 - 28°C)/8 h dark (22 - 25°C) photoperiod.

The T-DNA insertion mutants *jid1-1* (SALK_092918) and *jid1-5* (SALK_095793) were obtained from Nottingham *Arabidopsis* Stock Centre (NASC). The homozygous plants were selected by PCR using T-DNA primers. The *coi1-1* mutant was described previously[1]. To generate *p35S::Flag-JID1* (*JID1-OE*) or *p35S::JID1-GFP (JID1-GFP)* transgenic plants, the full-length coding sequence (CDS) of *JID1* was amplified and inserted into the *pCAMBIA1300-nFlag* or *pCAMBIA1300-221-cGFP* vector controlled by the *CaMV 35S* promoter, respectively. To generate *pJID1::GUS* transgenic plants, 1.9 kb *JID1* promoter genomic sequence was amplified and inserted into the *pCAMBIA1391z-GUS* vector to drive GUS expression. The *Agrobacterium* strain GV3101 carrying the *p35S::Flag-JID1*, *p35S::JID1-GFP*, or *pJID1::GUS* construct was transformed into the *Arabidopsis* WT plants by floral dip method[2], respectively. The transformed seedlings were screened on Murashige and Skoog (MS, Sigma-Aldrich) plates containing 50 mg/L hygromycin. Primers were presented in Supplementary Table S1.

Plant treatment, RNA extraction, and real-time PCR

The 7-day-old seedlings were treated with ethanol solvent, 15 μM OPDA, 25 μM MeJA, or 50 μM MeJA for indicated time, respectively. The fully-expanded

7th, 8th, and 9th leaves of 4-week-old plants grown in the SD photoperiod were wounded by the hemostat as described previously[3]. These wounded leaves were collected at the indicated time points. Total RNA extraction and real-time PCR analysis were performed following the manufacturer's instructions (Applied Biosystems). The *ACTIN2* (*AT1G49240*) was used as the internal reference. Primers were presented in Supplementary Table S1.

Semiquantitative RT-PCR

The semiquantitative RT-PCR of *JID1* was performed with Q5 polymerase (New England Biolabs). PCR conditions were 98°C 3 min, 25 cycles of 98°C 10 s, 50°C 30 s, 72°C 45 s. The *ACTIN8* (*AT3G18780*) was used as the internal reference. All PCR products were verified by sequencing. Primers were presented in Supplementary Table S1.

Protein extraction and western-blot

Total protein of plants was extracted with RB buffer [50 mM Tris-HCl (pH 7.8), 100 mM NaCl, 10% (v/v) glycerol, 0.1% (v/v) Tween-20, 20 mM β-mercaptoethanol] and quantified using the Bradford method. The absorbance values of 595 and 450 were determined to calculate plant protein concentration. After addition of 5 × SDS loading buffer, the protein samples were boiled at 95°C for 5 min. Then western-blot was performed for the detection and analysis of JID1 proteins.

GUS staining

The T2 transgenic seedlings or plants of *pJID1::GUS* were incubated in X-Gluc buffer [0.2 mM potassium ferricyanide, 0.2 mM potassium ferrocyanide, 1 mM EDTA (pH 8.0), 0.5% TritonX-100, 1 mM X-Gluc] in darkness at 37°C overnight. The histochemically-stained seedlings or plants were cleared by 75% ethanol. Then, the GUS staining patterns of seedlings or plants were observed and photographed.

Subcellular localization analysis

The *pCAMBIA1300-221-cGFP*, *p35S::JID1-GFP*, and *mCherry-PTS1* plasmids were transformed into *Agrobacterium* strain GV3101 respectively, and then introduced into *N. benthamiana* leaves. Before imaging, the leaves were injected with 20 µg/mL DAPI solution for 40 min. The T2 transgenic seedlings of *p35S::JID1-GFP* were used for fluorescence detection. The GFP fluorescence, DAPI, chloroplast autofluorescence, or mCherry fluorescence was detected by a confocal microscope (Olympus FV1200) with an excitation and emission of 488 - 525 nm, 358 - 461 nm, 488 - 550 nm, or 561 - 600 nm, respectively.

Nucleocytoplasmic fractionation

Plant cells were lysed with the extraction buffer containing 10 mM Tris-HCl (pH 7.5), 10 mM NaCl, 10 mM $MgCl_2$, 10% (v/v) glycerol, 10 mM β-mercaptoethanol, and 1 × protease inhibitor cocktail. The resulting lysates was spun through a 95 µm nylon mesh at 1,500 *g*, 4°C for 3 min. Then the filtrate was centrifuged at 3,000 *g*, 4°C for 12 min to pellet the nuclei. The supernatant was collected as the cytosolic fraction by recentrifugation at 13,000 *g*, 4°C for 15 min.

Pelleted material was washed by washing buffer [10 mM Tris-HCl (pH 7.5), 10 mM NaCl, 10 mM $MgCl_2$, 1 M hexylene glycol, 0.5% (v/v) Triton X-100, and 10 mM β-mercaptoethanol], and then incubated in the extraction buffer to use as the nuclear fraction[4].

Heterogeneous expression of JID1 in *E. coli* system

The full-length CDS fragment of *JID1* was inserted into the *pGEX6p-1* vector with an N-terminal GST tag and verified by sequencing. Then the plasmid was transformed into *E. coli* BL21 (DE3). After purification by Glutathione Sepharose 4B (GE Healthcare) affinity chromatography, JID1 was released by on-column cleavage to remove GST tag, and then further purified by Hitrap Q (GE Healthcare) followed by Superdex 200 10/300 (GE Healthcare) in the

buffer containing 10 mM Tris-HCl (pH 7.5), 100 mM NaCl, 5 mM DTT[5].

JA quantification *in vivo* by LC-MS/MS

The leaves of 4-week-old plants grown in the SD photoperiod were harvest at 0 h and 1 h after wounding. JA extraction and quantification by LC-MS/MS were performed as previously described[6] with an AB Sciex 4500 QTRAP triple quadrupole mass spectrometer (AB SCIEX) equipped with an ACQUITY UPLC™ BEH C18 column (Waters) (50 × 2.1 mm, 1.7 μm). Solvents for the mobile phase were 0.1% formic acid in acetonitrile (A) and 0.1% formic acid in acetonitrile (B). The gradient elution was 0 - 10 min, linear gradient 50 - 100%. Multiple reaction monitoring (MRM) mode was applied for quantification, the MRM transitions for JA were: 214.1494 > 62.0322 for d_5-JA, 291.1962 > 165.1280 for OPDA, 209.1180 > 59.0134 for jasmonic acid, 322.2020 > 130.0087 for JA-Ile. Mass spectrometry data were acquired and processed by AB SCIEX analyst 1.6.3 software (Applied Biosystems).

Purification of transiently expressed JID1 protein in *N. benthamiana* system and enzyme activity assay

The 4-week-old *N. benthamiana* leaves were inoculated by *Agrobacterium* strain GV3101 carrying an empty vector (*pCAMBIA1300-nFlag*) or *p35S::Flag-JID1*. The purification of transiently expressed JID1 was performed as a previously described method[7]. The enzyme activity assay was performed as described with some modifications[8]. The incubation system was 50 mM Tris-HCl (pH 7.5), 5 mM DTT, 8 mM α-ketoglutarate, 8 mM ascorbic acid, 1 mM $FeSO_4$, 25 μM substrate, 10 μg purified protein complemented with 2 mg/mL BSA. The reaction was incubated in darkness at 30°C for 4 h and stopped by adding 1 mL 100% methanol. After centrifugation at 13,000 *g*, 4°C for 10 min, JA (OPDA, jasmonic acid, MeJA, or JA-Ile) in the supernatant was analyzed by a Dionex Ultimate 3000 UHPLC system coupled with a TSQ Quantiva Ultra triple-quadrupole mass spectrometer (Thermo Fisher, CA, USA) heated

electrospray ionization (HESI) probe. Extracts were separated by an ACQUITY UPLC™ BEH C18 column (Waters) (100 × 2.1 mm, 1.7 μm). Solvents for the mobile phase were 0.1% formic acid in 100% H_2O (A) and 0.1% formic acid in acetonitrile (B). A 10-minute gradient with flow rate of 300 μL/min was used as follows: 0 - 1.5 min at 15% B; 1.5 - 5min, 15 - 98% B; 5 - 7 min, 98% B; and 7.1 - 10 min, 15% B. MRM mode was used for target compounds analysis (214.1494 > 62.0322 for d_5-JA, 291.1962 > 165.1280 for OPDA, 209.1180 > 59.0134 for jasmonic acid, 322.2020 > 130.0087 for JA-Ile under negative mode; 225.1491 > 151.1123 for MeJA under positive mode). Data analysis and quantification were processed by TraceFinder analysis software (version 3.3, Thermo Fisher Scientific Inc., Waltham, MA, USA).

OPDA and mo-OPDA detection *in vitro* by LC-MS/MS

The substrate (OPDA) and product (mo-OPDA) were detected by the UHPLC system coupled with a Q-Exactive orbitrap mass spectrometer (Thermo Fisher, CA, USA) equipped with a HESI probe. Extracts were separated by an ACQUITY UPLC™ BEH C18 column (Waters) (100 × 2.1 mm, 1.7 μm) under both positive and negative (ESI+ and ESI-) modes.

Solvents for the mobile phase were 5 mM Ammonium acetate in H_2O (A) and 5 mM Ammonium acetate in acetonitrile (B). A 10-minute gradient same as the above section with flow rate of 300 μL/min was used. Data with mass ranges of *m/z* 80 - 1200 and *m/z* 70 - 1050 were acquired at both positive and negative ion modes with data dependent MS/MS acquisition. The full scan and fragment spectra were collected with the resolution of 70,000 and 17,500, respectively. The source parameters were as follows: spray voltage: 3000 V; capillary temperature: 320°C; heater temperature: 300°C; sheath gas flow rate: 35 Arb; auxiliary gas flow rate: 10 Arb. Then data were analyzed with TraceFinder analysis software (version 3.3, Thermo Fisher Scientific Inc., Waltham, MA, USA).

B. cinerea infection and defense response assay

The *B. cinerea* strain B05.10 was inoculated into potato dextrose broth medium under 12 h light/12 h dark photoperiod at 20°C for 10 d. The *B. cinerea* infection assay and the *Arabidopsis*/*B. cinerea* genomic DNA extraction were performed as previously described[9,10].

For whole-plant spray inoculation, *B. cinerea* spore suspension (10^5 spores/mL) was sprayed on the 3-week-old plants grown in the SD photoperiod. Plants were cultured under consistent wet or humid conditions for 3 - 4 d, then disease severity is categorized as follows: leaf necrosis rate less than 30% (light) is marked as green; leaf necrosis rate of 30 - 70% (moderate) is marked as light brown; leaf necrosis rate more than 70% (severe) is marked as dark brown.

The infected plants were collected for extraction of the *Arabidopsis*/*B. cinerea* genomic DNA with DNA extraction buffer [0.1 M Tris-HCl (pH 8.8), 0.05 M EDTA (pH 8.0), 0.5 M NaCl, 2% SDS]. The extracted genomic DNA was used for real-time PCR, the abundance of *B. cinerea* biomass was quantified using the relative ratios of *B. cinerea* genomic *CUTINASE* (*CUT*) DNA versus *Arabidopsis* genomic *ACTIN2* DNA. Primers were presented in Supplementary Table S1.

S. exigua feeding and defense response assay

The *S. exigua* eggs (2nd instar) were placed in a culture dish covered with water-soaked filter paper and hatched at 28°C for about 2 d. Then, newly hatched larvae were transferred into a new culture dish with solid insect feed at 28°C for 3 - 4 d. Ten *S. exigua* larvae were placed on rosette leaves of four 4-week-old plants grown in SD photoperiod for 3 d. Larval weight and phenotypes were measured and photographed, respectively.

1. Xie D, et al. Science **280**, 1091-1094 (1998).
2. Clough SJ & Bent AF. Plant J. **16**, 735-743 (1998).
3. Li M, et al. Mol. Plant **13**, 1485-1498 (2020).
4. Genenncher B, et al. Plant Physiol. **172**, 1293-1305 (2016).

5. Yao R, et al. Cell Res. **27**, 838-841 (2017).
6. Glauser G & Wolfender JL. Methods Mol. Biol. **1011**, 123-134 (2013).
7. Yan J, et al. Plant Cell **25**, 486-498 (2013).
8. Caarls L, et al. Proc. Natl. Acad. Sci. U. S. A. **114**, 6388-6393 (2017).
9. Hu P, et al. Mol. Cell **50**, 504-515 (2013).
10. Smirnova E, et al. Mol. Plant **10**, 1159–1173 (2017).
